# Differential expression of genes in the alate and apterous morphs of the brown citrus aphid, *Toxoptera citricida*

**DOI:** 10.1038/srep32099

**Published:** 2016-08-31

**Authors:** Feng Shang, Bi-Yue Ding, Ying Xiong, Wei Dou, Dong Wei, Hong-Bo Jiang, Dan-Dan Wei, Jin-Jun Wang

**Affiliations:** 1 Key Laboratory of Entomology and Pest Control Engineering, College of Plant Protection, Southwest University, Chongqing 400716, China

## Abstract

Winged and wingless morphs in insects represent a trade-off between dispersal ability and reproduction. We studied key genes associated with apterous and alate morphs in *Toxoptera citricida* (Kirkaldy) using RNAseq, digital gene expression (DGE) profiling, and RNA interference. The *de novo* assembly of the transcriptome was obtained through Illumina short-read sequencing technology. A total of 44,199 unigenes were generated and 27,640 were annotated. The transcriptomic differences between alate and apterous adults indicated that 279 unigenes were highly expressed in alate adults, whereas 5,470 were expressed at low levels. Expression patterns of the top 10 highly expressed genes in alate adults agreed with wing bud development trends. Silencing of the lipid synthesis and degradation gene (3-ketoacyl-CoA thiolase, mitochondrial-like) and glycogen genes (Phosphoenolpyruvate carboxykinase [GTP]-like and Glycogen phosphorylase-like isoform 2) resulted in underdeveloped wings. This suggests that both lipid and glycogen metabolism provide energy for aphid wing development. The large number of sequences and expression data produced from the transcriptome and DGE sequencing, respectively, increases our understanding of wing development mechanisms.

Many insect species have dispersing and non-dispersing morphs. These include short-winged and long-winged morphs in planthoppers^1^, crickets^2^, migratory locusts^3^, and wingless and winged morphs in aphids^4^,^5^. These phenotypes are associated with different dispersal capabilities. Typically, alate and apterous morphs develop in response to specific environmental conditions^6^. Wing dimorphism is clearly an adaptation to fluctuating environmental conditions, increasing the dispersal capability of the insects and allowing them to escape unfavorable conditions^6^. Many studies have focused on the abiotic and biotic factors influencing aphid wing dimorphism. For instance, increased population density triggers wing formation in most aphid species and in some species, a relatively small density increase is sufficient^7^,^8^,^9^. A decrease in plant quality can trigger wing induction in the bird cherry-oat aphid, *Rhopalosiphum padi*^10^. Relatively higher temperatures favor wingless forms while lower temperatures facilitate wing induction. Studies on starvation combined with symbiosis indicate a significant role of symbionts in wing dimorphism of the English grain aphid, *Sitobion avenae*^11^.

Wing dimorphism represents a life history trade-off between dispersal and reproduction^1^,^12^,^13^,^14^. In aphids, alate and apterous morphs differ in presence or absence of wings and also differ in body structure, fecundity, longevity and behavior. Flight morphs have heavier sclerotization of the head and thorax, more fully developed compound eyes and ocelli, longer antennae, more rhinaria, and sometimes larger siphunculi and cauda^6^,^15^,^16^. The morphological differences between apterous and alate morphs usually correlate with differences in life history. In general, the alate morph, compared to the apterous morph, has a longer nymphal development period, longer pre-reproductive adult period, lower offspring production, and greater longevity^17^,^18^. Alate morphs are also more resistant to starvation and they have acute sensory capability for detecting host plants^19^.

The brown citrus aphid, *Toxoptera citricida* (Kirkaldy) (Hemiptera: Aphididae), is an important citrus pest and the main vector of *Citrus tristeza virus* (CTV) worldwide. Like other aphids, *T. citricida* has wing dimorphism. The apterous morph is about 3.2 mm in body length, is shiny black, lacks wing remnants^20^,^21^, and possesses higher fecundity. The alate morph is smaller than the apterous morph, has 5-mm-long wings^20^,^21^, and strong flight muscles. This allows it to fly long distances with the wind and to spread CTV in citrus growing regions. These differences are likely associated with the gene expression of alate and apterous morphs. Unlike the pea aphid, *Acythosiphon pisum*^22^, and Russian wheat aphid, *Diuraphis noxia*^23^, the genome of *T. citricida* has not been sequenced or released. Hunter *et al*.^24^ completed expressed sequence tag (EST) sequencing of alate *T. citricida*. About 5,180 cDNA clones were sequenced resulting in 4,263 ESTs^24^, but this was insufficient for gene identification and prediction of functions. Fortunately, the emergence of next-generation sequencing (high-throughput sequencing) technology has dramatically enhanced the efficiency and quantity of gene annotation. In insects, transcriptome sequencing combined with digital gene expression (DGE) analysis is a reliable and precise way to investigate transcriptomic characteristics, such as insecticide targets^25^, immune response^26^, and chemoreception^27^. RNA interference (RNAi) methods have been used to study the function of genes involved in insect molting, growth, and development^28^,^29^,^30^. Thus, we expected that the transcriptome and DGE analysis of alate and apterous adults combined with RNAi would greatly improve understanding of molecular level differences between alate and apterous morphs, especially for the genes associated with dispersion of the alate morph. Here we reported: (1) transcriptome analysis covering different developmental stages of *T. citricida*, (2) DGE profiling of alate and apterous adults, (3) validation of the fold change of the top ten highly expressed genes in alate adults by quantitative real-time PCR (qRT-PCR) and examination of gene expression levels in different developmental stages, wing morphs, and body parts, and (4) functional analysis, using RNAi, of the energy metabolism genes which may be involved in wing development. The results provide a resource for future functional studies on wing dimorphism and development of *T. citricida* and other aphids.

## Results

### Transcriptome sequencing and assembly

A library of different developmental stages of *T. citricida* was developed by Illumina sequencing in a single run, which generated 41,250,684 raw reads, containing about 8.33 Gb sequencing data. After initial adaptor trimming and quality filtering, 1,199,882 contigs with an N50 length of 101 bp were assembled from 37,182,535 clean reads. The sequencing quality of the clean reads was evaluated based on the base-calling quality scores of Illumina’s base-caller Bustard software. More than 96% of the clean reads had quality scores higher than the Q_30_ level (an error probability of 0.1%) (see Supplementary Fig. S1). The contigs were further assembled into 44,199 unigenes with a mean length of 749 bp by using paired-end joining and gap-filling methods (Table 1). Among the total unigenes, 9,468 unigenes (21.4%) were longer than 1,000 bp and 26,443 unigenes (58.8%) were among 200 bp to 500 bp, and no unigene was less than 200 bp (see Supplementary Fig. S2).

### Annotation of predicted transcripts

**Figure 1:**
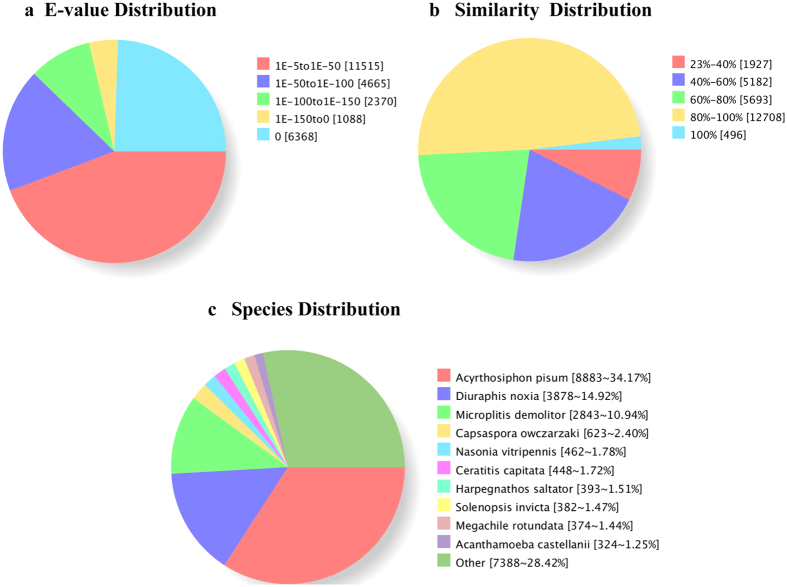
Homology analysis of unigenes of *Toxoptera citricida*. (**a**) E-value distribution of BLAST hits for each unigene with a cut-off of 1.0E-5; (**b**) Similarity distribution of the top BLAST hits for each sequence; (**c**) Species distribution. The species distribution is shown as the percentage of the total homologous sequences in the NCBI Nr protein database with an E-value < 10-5.

Unigene sequences were annotated using BLASTX against the non-redundant (NR) NCBI protein database. The E-value distribution of the annotated unigenes showed that 53.5% of the sequences had high homology (<1.0 E-50), while 46.5% of the sequences ranged between 1.0 E-5 to 1.0 E-50 (Fig. 1a). Further analysis of the similarity distribution indicated that 50.8% of the sequences had similarity higher than 80%, 21.9% of the sequences with a similarity between 60% to 80%, and only 7.4% of the sequences possessed a similarity lower than 40% (Fig. 1b). According to the best hits in the NR database, about half of annotated unigenes (12,761 unigenes, 49.1%) exhibited homology with the pea aphid, *A. pisum*, and Russian wheat aphid, *D. noxia*, which reflected the close relationship among *T. citricida*, *A. pisum* and *D. noxia,* while 28.4% of them matched those in other species (Fig. 1c).

### Unigene function annotation and metabolic pathway analysis

Assignments of Clusters of Orthologous Groups (COG) were used to predict and classify possible functions of the unigenes. Based on sequence homology, 11,052 unigenes were annotated and divided into 26 specific categories (see Supplementary Fig. S3). The general function category, which contained 2,759 unigenes (25.0%), was the largest, followed by translation, ribosomal structure, and biogenesis (1,283, 11.6%), replication, recombination and repair (1,242, 11.2%), and transcription (1,196, 10.8%), respectively. Only 1 unigene (less than 1%) belonged to extracellular structures, which was the smallest group.

For Gene Ontology (GO) analysis, we categorized 12,189 unigenes into 59 function groups. Among these, metabolic process, cellular process, and catalytic activity were the 3 largest groups, containing 8,254, 7,361, and 7,164 unigenes, respectively. Only 1 unigene each was predicted to act in the component and function groups including protein tag, nutrient reservoir activity, morphogen activity, cell death, and carbon utilization (see Supplementary Fig. S4).

We further mapped 10,522 unigenes to 202 pathways in the Kyoto Encyclopedia of Genes and Genomes (KEGG) annotation system. Metabolic pathways containing 2,180 unigenes (20.7%) were significantly larger than other pathways, such as protein processing in the endoplasmic reticulum (446, 4.2%), RNA transport (415, 3.9%), and spliceosome (403, 3.8%; see Supplementary Table S1).

### Summary of digital gene expression sequencing

DGE analysis was performed to obtain a global view of the aphid transcriptome differences alate and apterous adults. To compare differentially expressed genes between the libraries (alate/apterous), the level of gene expression was determined by normalizing the number of unambiguous tags in each library to reads per kilobase mapped (RPKM). DGE gave high reproducibility among biological replicates, as indicated by the Pearson correlation coefficient (*r*) ≥ 0.98 (Alate1 versus Alate 2: 0.9925, Apterous 1 versus Apterous 2: 0.9881; see Supplementary Fig. S5). In comparison with apterous adults, the expression levels of 5,749 unigenes was significantly different in alate adults (see Supplementary Fig. S6) including 279 genes with higher expression (see Supplementary Table S2) and 5,470 genes with lower expression (see Supplementary Table S3). The fold changes (log_2_Ratio) was calculated based on the gene expression level of alate divided by that of apterous adults.

Using the GO classification, these differentially expressed genes were characterized into 3 groups: biological process, cellular component and molecular function (see Supplementary Fig. S7). Among the biological process assignments, a high percentage of genes were concentrated in the cellular process category (1,024 genes, 19.2%), predominantly in the microtubule-based movement, spindle organization, and metabolic process category (969 genes, 18.2%). The cellular component group showed a significant percentage of genes assigned to cell part (1,074 genes, 25.3%) and organelle (737 genes, 17.3%) categories, where they were mainly concentrated in the microtubule dynein complex and mitochondrial respiratory chain.

KEGG pathway analysis of the highly expressed transcripts in alate adults was performed. Among the highly expressed transcripts, most genes could be annotated in KEGG orthology (KO) based on sequence homologies and mapped to 60 pathways, and the top 20 enriched metabolic pathways are listed in Table 2. Pathway-oriented analysis showed that most pathways were involved in energy resources and nutrient-sensing pathways, such as the citrate cycle (TCA cycle), fatty acid metabolism, fatty acid elongation, insulin signaling pathway, glyoxylate and dicarboxylate metabolism, and glycolysis/gluconeogenesis.

### qRT-PCR validation

To confirm the DGE results by Illumina sequencing, we selected the top 10 highly expressed genes in alate adults (Sodium-and chloride-dependent creatine transporter 1-like isoform 1 (*SCDCT*), 3-ketoacyl-CoA thiolase, mitochondrial-like (*KAT*), ATP-binding cassette sub-family G member 4-like isoform 1 (*ABCG4*), trifunctional enzyme subunit beta, mitochondrial-like (*β-TE*), short-chain specific acyl-CoA dehydrogenase, mitochondrial-like (*SCAD*), trifunctional enzyme subunit alpha, mitochondrial-like (*α-TE*), phosphoenolpyruvate carboxykinase [GTP]-like (*PEPCK*), hydroxysteroid dehydrogenase-like protein 2-like (*HSD*), E3 ubiquitin-protein ligase TRIM23-like (*E3-TRIM23*), glycogen phosphorylase-like isoform 2 (*GP*) for qRT-PCR confirmation. Their annotations and fold changes are summarized in Table 3. All 10 genes showed similar change trends as indicated by the DGE analysis (see Supplementary Fig. S8), suggesting that the DGE approach can be a powerful tool for the discovery of candidate genes in a high-throughput manner. However, there were some fold change magnitude differences between qRT-PCR experiments and DGE analysis. For example, we found 37-fold in qRT-PCR versus 8-fold in DGE for *SCDCT* and 16-fold in qRT-PCR versus 5-fold in DGE for *PEPCK*.

The expression patterns of the top 10 highly expressed genes in alate adults were further determined in different developmental stages, wing morphs, and body parts, respectively.

**Figure 2:**
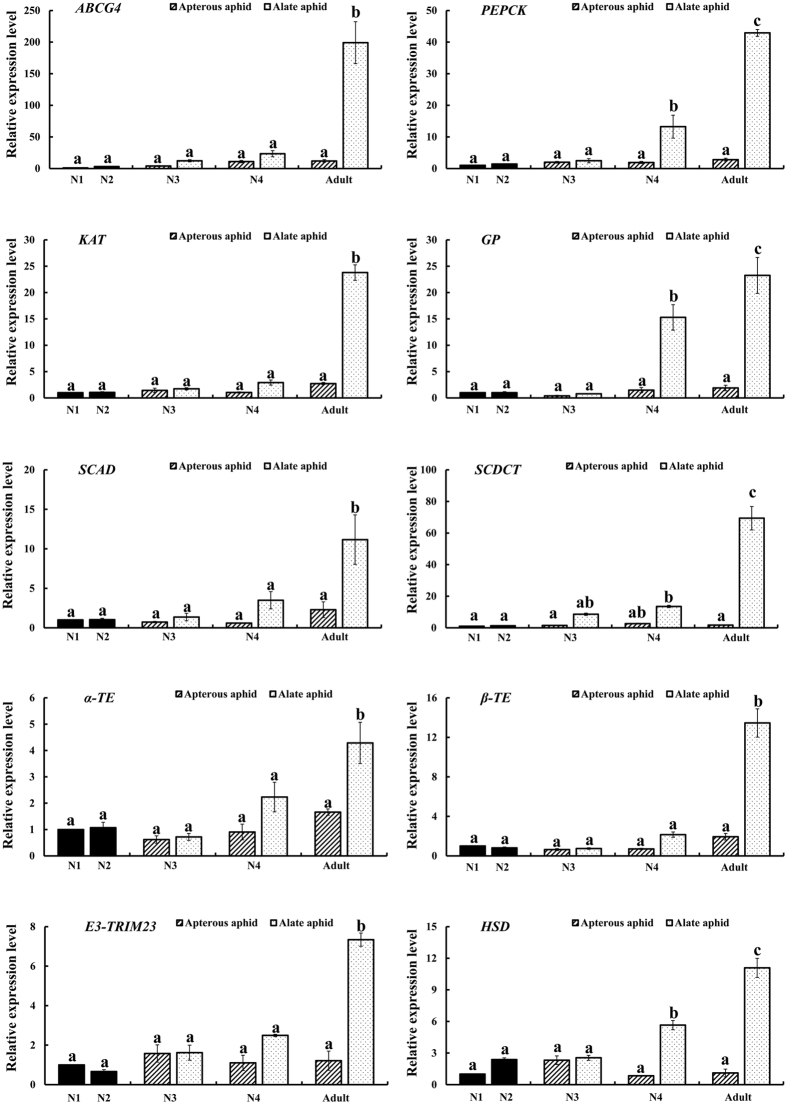
Relative expression levels of top 10 highly expressed genes in alates in different developmental stages of *Toxoptera citricida*. N1, first instar nymphs; N2, second instar nymphs; N3, third instar nymphs; N4, fourth instar nymphs. Bars with a slash line indicate apterous aphids and bars with scatter indicate alate aphids in each stage, apterous and alate aphids cannot be distinguished by examining morphology of first and second instar nymphs. Data are means ± SE of 4 biological replicates and the elongation factor-1 alpha (*EF1α*) was used as a reference gene. Lowercase letter (**a–c**) above each bar indicates a significant difference among different developmental stages and wing morphs using one-way ANOVA followed by Tukey’s honestly significant difference test (*P* < 0.05). The relative expression was calculated using the 2^−△△*Ct*^method based on the value of the first instar nymph expression, which was ascribed an arbitrary value of 1.

All gene expression levels were sharply increased from fourth instar nymphs to adults and most showed no difference among the nymphal stages of alates. No significant differences existed among nymphal stages and adult in apterous aphids (Fig. 2). The wing bud developed slowly in each nymphal instar until the fully formed wings unfolded after adult emergence. This means that the fourth instar nymph to adult transition was the key period of aphid wing development. The gene expression patterns agree with the trend in wing development. Thus, these genes might be involved in wing development.

**Figure 3:**
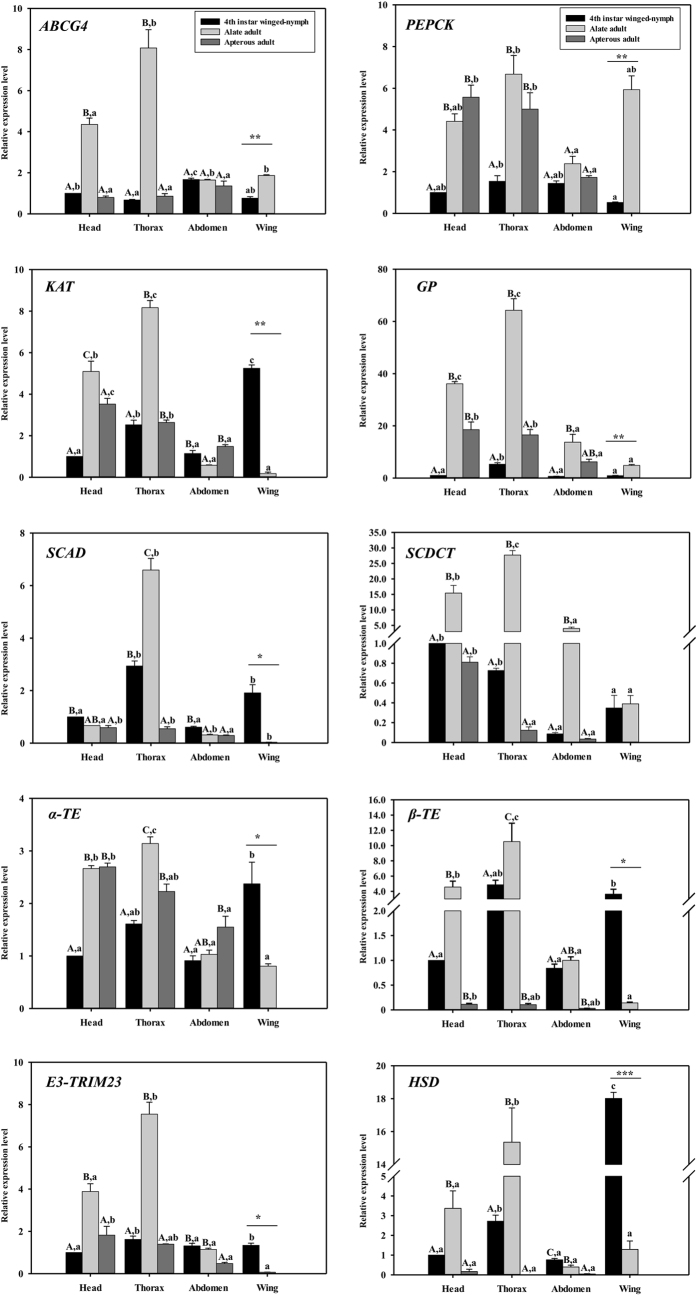
Relative expression levels of the top 10 highly expressed genes in alate adults in different body parts of *Toxoptera citricida*. The relative expression was calculated using the 2^−△△*Ct*^method based on the value of the head of fourth instar winged-nymphs expression, which was assigned an arbitrary value of 1. Uppercase letter A, B, and C above each bar indicates a significant difference among different developmental stages and wing morphs within same body parts using one-way ANOVA followed by Tukey’s honestly significant difference test (*P* < 0.05). Lowercase letter a, b, and c above each bar indicates a significant difference among different body parts within same developmental stages and wing morphs using one-way ANOVA followed by Tukey’s honestly significant difference test (*P* < 0.05). A significant difference of gene expression level in wing/wing bud between fourth instar winged-nymphs and alate adults is indicated with asterisks (*P < 0.05; **P < 0.01; ***P < 0.001, Student’s *t* test).

Head, thorax, abdomen and wing were detached from fourth instar winged-nymphs, alate adults and apterous adults for expression pattern analyses. The results showed that all the genes were highly expressed in the thorax, and about 5-fold greater compared to other body parts of *SCAD* and *HSD* in alate adults. The changes were different for wing-related genes between fourth instar winged-nymphs and alate adults. *ABCG4*, *PEPCK*, *GP* were highly expressed in alate adults but *KAT*, *SCAD*, *α-TE*, *β-TE*, *E3-TRIM23*, and *HSD* exhibited higher expression levels in fourth instar winged-nymphs (Fig. 3), suggesting the potential crucial roles of the genes in wing development.

### Functional analyses

**Figure 4:**
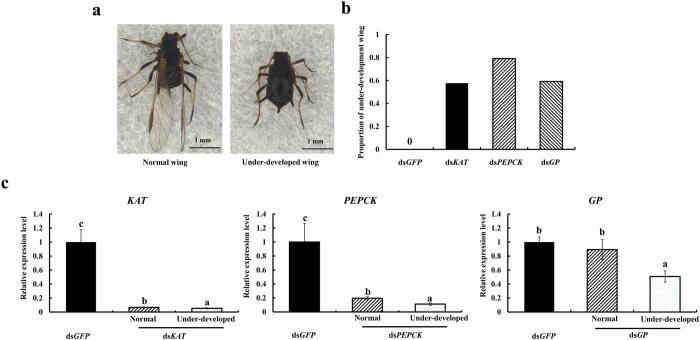
dsRNA feeding mediated RNAi and its effects on wing development of *Toxoptera citricida*. (**a**) Representative phenotypes of *Toxoptera citricida* after feeding with dsRNA of *KAT*, *PEPCK*, and *GP*. (**b**) The rate of under-developed wing aphid after dsRNA feeding at 72 h. (**c**) Relative expression level of *KAT*, *PEPCK*, and *GP* after RNAi. The relative expression was calculated using the 2^−△△*Ct*^method based on the value of ds*GFP* (control) at 72 h that was ascribed an arbitrary value of 1. Data are means ± SE of 4 replicates and the elongation factor-1 alpha (*EF1α*) was used as a reference gene. Lowercase letters above each bar indicate significant differences among different samples using one-way ANOVA followed by Tukey’s honestly significant difference test (*P* < 0.05).

RNAi was used to study the lipid synthesis/degradation gene (*KAT*) and glycogen related genes (*PEPCK* and *GP*), and their effects on wing development. Aphids were synchronized as new fourth instar winged-nymphs and fed with dsRNA. After 72 h, all aphids normally molt from fourth instar nymphs to adults, but RNAi treated aphids showed a variety of different phenotypes including those with under-developed, malformed, and normal wings. Because both malformed and under-developed wing aphids were unable to fly and the malformed wing aphid appeared at a very low rate (<5%), we categorized this type into the under-developed wing when counting the phenotype and collecting samples (Fig. 4a). Silencing of these 3 genes resulted in under-developed wing aphids at rates of 58% (ds*KAT*) to 79% (ds*PEPCK*) compared to ds*GFP*, which was 100% normal (Fig. 4b). When challenged at 72 h, the relative expression levels of these 3 genes in under-developed wing aphids were down-regulated by 50% (*GP*), 74% (*PEPCK*), and 95% (*KAT*). The relative expression levels of *KAT* (93%), *PEPCK* (75%) were decreased in normal wing aphids by feeding with dsRNA compared to ds*GFP* but the difference was not significant in the ds*GP* treatment (Fig. 4c). These results indicate that the gene expression levels in under-developed wing aphids were all lower than normal when the aphids were treated with dsRNA.

## Discussion

Flight capacity in insects is a key feature behind their ecological and evolutionary success. Flight benefits dispersal capacity balanced against potential metabolic, reproductive and survival costs^14^,^31^. In this study, we used the transcriptomic tools including RNA-seq and DGE, and RNAi technology, to analyze the molecular mechanisms of wing development of *T. citricida*.

We generated *de novo* assembly of the *T. citricida* transcriptome through short read sequencing technology (Illumina). In a single run, we obtained more than 41 million sequencing reads that were assembled into 44,199 unigenes by Trinity. Homology analysis of the unigenes demonstrated that only 49.1% showed the greatest similarity to *A. pisum* and *D. noxia*, the only 2 aphid species with a completely sequenced genome^22^,^23^. This rate is smaller than expected. *A. pisum* and *D. noxia* belong to Macrosiphini, but *T. citricida* belongs to Aphidini, and the host range of *T. citricida* is limited to citrus and a few close relatives^32^,^33^. The new transcriptome data reported here complements the scarce aphid sequence resources in the GenBank. We found that 12.3% of unigenes matched those in *Microplitis demolitor* and *Nasonia vitripennis*, which are endoparasitoids of Lepidopteran and Dipteran^34^,^35^, and we also found that 1.5% of the unigenes matched those in the fire ant *Solenopsis invicta*. Our laboratory will be focusing on this interesting phenomenon to explore the potential horizontal gene transfer among endoparasitoids, aphids, and ants.

The transcriptomic differences between alate and apterous adults were examined by DGE to explore the molecular mechanism associated with wing morphs, especially the genes influencing the development or behavior of alates. Through DGE analysis, we produced a total of 5,749 differentially expressed transcripts, of which 279 transcripts were highly expressed in alate adults. The highly expressed transcripts in alate adults belonged to 60 KEGG pathways, and most of the pathways were involved in energy resource and nutrient-sensing. The insulin signaling pathway was interesting pathway owing to its role in energy distribution, and this is an evolutionarily conserved nutrient-sensing pathway that modulates growth and development in metazoans^36^,^37^. In rice brown planthopper, *Nilaparvata lugens*, silencing of 2 insulin receptor genes (*NlInR1* and *NlInR2*) by RNAi was used to understand the regulation of wing dimorphism by the insulin/insulin-like signaling pathway^38^. This was the first evidence of a molecular basis for the regulation of wing polyphenism in a planthopper. The current results also implied that insulin/insulin-like signaling pathway maybe play an crucial role in alate aphid.

The qRT-PCR experiment confirmed the DGE results. All of the top 10 highly expressed genes in alate adults subjected to the qRT-PCR analysis showed changes similar to the DGE results but the fold differences varied. The discrepancies in the fold changes between qRT-PCR and DGE may have resulted from a sensitivity bias between the 2 methods or by virtue of different statistical methods and threshold values in qRT-PCR and DGE^39^. The analyses of gene expression patterns showed that almost all genes had low expression levels in nymphal stages but sharply increased in the transition from fourth instar winged nymphs (N4-WD) molting to alate adults. This result was consistent with findings that the transition period between the final instar winged-nymph to alate adults is the key stage of wing development^40^. The results indicated that these genes may be involved in wing development, and this was subsequently verified by dsRNA feeding mediated RNAi. Further analysis of gene expression levels among different body parts of N4-WD, alate, and apterous adults showed that all of the genes had their highest expression level in thorax except *HSD* suggesting that these genes may be involved in the formation or development of flight muscle^41^,^42^. We also found that gene expression differed in wings of N4-WD and alate adults, suggesting that they may play positive or negative roles in wing development^30^.

Of the 279 highly expressed unigenes in alate adults, the top 10 highly expressed genes were chosen to clarify their potential functions. *KAT*, *SCAD*, *α-TE*, and *β-TE* were “mitochondrial like” genes that may contribute to energy production and are thought to be involved in aspects of the citric acid cycle respiratory chain, ATP synthesis, and transport^5^. In *Aphis gossypii* and *A. pisum*, very long-chain specific, medium-chain, and short-chain specific acyl-CoA dehydrogenase, and *KAT* were highly expressed in alate adults. These genes, associated with lipid synthesis and degradation and genes involved in sugar metabolism are elevated in apterous adults, indicating that the aphids use lipid rather than sugar or glycogen as fuel during flight^5^,^43^. Of particular interest is that, in addition to the lipid synthesis/degradation related gene (*KAT*), *PEPCK*, and *GP* were also highly expressed in alate adults and these are linked with glycogenolysis. RNAi was further used to explore the function of lipid synthesis/degradation and glycogen related genes and their effects on aphid wing development. Silencing of *KAT*, *PEPCK*, and *GP* all resulted in under-developed wings and flight disruption of *T. citricida*. We found that the relative expression levels of *KAT* and *PEPCK* were significantly decreased in normal alates when challenged with dsRNA of target genes compared to ds*GFP*, and the relative expression levels of the target genes were also significantly different between alates with normal wings or under-developed wing for dsRNA treatments. One possible explanation is that the development period may vary among individuals from the fourth instar winged-nymph to the alate adult and the key period for wing development also may vary. Thus, although the genes were silenced, the phenotype remained unchanged. Another possibility is that there exists feedback regulation so when expression of *KAT* or *PEPCK* is decreased, the other gene might be able to keep the wing normal but when gene expression decreased below a certain level, this feedback will fail to work. The results suggested that both lipid and glycogen provide resources for wing development and dispersion in *T. citricida*, but the relative importance of these in dispersion is unclear. The role of lipid and glycogen during migration should be studied. Additional functions of these genes or their functions in apterous morphs require more study.

In conclusion, this study is the first report of genetic information on *T. citricida* from sequenced transcriptome and constructed DGE libraries. The data revealed a large number of genes, with both known and unknown functions, which greatly enrich the sequence information of aphids. In addition, we also identified genes that are candidates related to wing development in *T. citricida*. The RNAi results predict the possible function of lipid synthesis/degradation and glycogen related genes in wing development.

## Methods

### Insect culture

*T. citricida* used in this study were established in 2012 from a single collection of wild alate aphids from a screen house at Southwest University, Chongqing, China. Stock colonies were maintained on potted citrus seedlings (*Citrus sinensis*) in the laboratory at 25 ± 1 °C, 75–80% relative humidity, and a photoperiod of 14: 10 h (Light: Dark). Alate morphs were induced by high-density aphid rearing after transfer to fresh host plants^9^,^24^. All progeny were produced by parthenogenesis from the stock colony.

### RNA isolation, cDNA library preparation and Illumina sequencing

Thirty first instar nymphs (N1), second instar nymphs (N2), third instar wingless and winged nymphs (N3-WL and N3-WD), fourth instar wingless and winged nymphs (N4-WL and N4-WD), apterous adults (AP, with mature embryos) and alate adults (AL, with mature embryos) were all collected at about 14:00 of the day for total RNA isolation, respectively. Apterous and alate adults were collected separately within 48 h after the fourth instar nymph molt. Total RNA was isolated with a TRIzol kit (Invitrogen, Carlsbad, CA) according to manufacturer instructions. RNA was quantified by measuring absorbance at 260 nm using a NanoVue UV-Vis spectrophotometer (GE Healthcare Bio-Science, Uppsala, Sweden). The purity of all RNA samples was assessed at an absorbance ratio of OD_260/280_ and OD_260/230_, and the integrity of RNA was checked on 1% agarose gel by electrophoresis. DNase I (Promega, Madison, WI) was used to remove genomic DNA from the samples.

The cDNA library construction and Illumina sequencing of the samples were performed at Beijing Biomarker Technologies, Beijing, China. Briefly, 12 μg of the RNA mixture (each development stages and wing morph were mixed at equal ratio) was used for cDNA library construction. Oligo (dT) magnetic beads were used to isolate poly (A) mRNA, which was then fragmented into small pieces by the addition of fragmentation buffer. These short fragments served as templates to synthesize first-strand cDNA using random hexamer-primers. Second-strand cDNA synthesis was performed using a buffer, dNTPs, RNaseH, and DNA polymerase I. Short fragments were purified using a QiaQuick PCR extraction kit (Qiagen) and then they were washed with ethidium bromide buffer for end preparation and single nucleotide adenine addition before being ligated to adaptors. Suitable fragments, as judged by agarose gel electrophoresis, were selected as templates for PCR amplification. The cDNA library was sequenced on Illumina Hiseq^TM^ 2000 using paired-end technology in a single run. Through sequencing, a total of 41,250,684 pair-end reads and 8,332,638,168 bases were achieved and the raw reads were submitted to the NCBI Short Read Archive (SRA) database with accession number of SRR2123649.

### *De novo* assembly and bioinformatics analysis

The raw reads were cleaned by removing adapter sequences, reads containing ambiguous bases, and low-quality reads. The sequencing quality of the clean reads was evaluated based on the base-calling quality scores of Illumina’s base-caller Bustard. The clean reads were assembled into contigs using the Trinity method (https://github.com/trinityrnaseq/trinityrnaseq/wiki), which is efficient in reconstructing full-length transcripts across a broad range of expression levels and sequencing depths with an optimized k-mer length of 25 for *de novo* assembly^44^. Then the transcripts were clustered based on nucleotide sequence identity. If there was more than 1 transcript for a given gene, the longest transcript was used to calculate its expression level and coverage.

All assembled unigenes were determined by BLASTx against the NCBI non-redundant (NR) protein database^45^, the Swiss-Prot database^46^, the Kyoto Encyclopedia of Genes and Genomes (KEGG) database^47^, Gene Ontology (GO)^48^ and Cluster of Orthologous Groups (COG)^49^ with a cut-off *E*-value of 10^−5^. If the alignment results of different databases conflicted with each other, the priority order of NR, Swiss-Prot, KEGG, GO, and COG were followed. When a unigene did not align with any of the entries in these databases, ESTs were used to predict its coding regions and to determine its sequence direction.

### DGE library preparation and sequencing

A total of 100 apterous or alate adults (2 biological replicates for each morph and 50 aphids in each biological replicate) were collected separately within 48 h after fourth instar nymphs molting at the same time of the day according to the above description. RNA extraction was done using TRIzol kit in according to manufacturer instruction, as described above, and DNase I (Promega, Madison, WI) was also used to remove genomic DNA from the samples. Approximately 30 μg RNA from each sample was used to construct the DGE libraries. The mRNA was treated as described in cDNA library construction and enriched by PCR amplification. The library products were then ready for sequencing analysis via Illumina Hiseq^TM^ 2000 (Beijing Genomics Institute, BGI, Shenzhen, China) using paired-end technology in a single run.

### Annotation of DGE tags and analysis of differentially expressed genes

All raw sequence reads were filtered using the Illumina pipeline before mapping reads to the reference transcriptome databases. Low-quality reads were omitted from data analysis. Low-quality reads were defined as those with the percentage of ambiguous bases >10% and reads in which >50% of the bases had a quality value ≤ 5. Clean reads were mapped to reference sequences (unigenes from the transcriptome data of different developmental stages was used as a reference) using SOAPaligner/soap2^50^. No more than 2 mismatches were allowed in the alignment. The level of gene expression was determined by normalizing the number of unambiguous tags in each sample to RPKM (reads per kilobase mapped)^51^. The relative gene expression in alate or apterous adult was equal to the mean of two biological samples of each morph. The fold change was calculated as fold change = log_2_Ratio. The differentially expressed transcripts between samples were identified using an Bayesian algorithm developed by Audic and Claverie^52^. The false discovery rate (FDR) method was used to determine the threshold of the *P*- value in multiple tests and analyses. The threshold FDR ≤ 0.001 and the absolute value of log_2_Ratio ≥ 1 were used to judge the significance of differences in gene expression. Then the genes that were expressed at different levels across the samples were further annotated by GO function and KEGG pathway analysis.

### Quantitative real-time PCR (qRT-PCR) analysis

To confirm the DGE results, the top 10 highly expressed genes in alate adults were chosen and the gene-specific primers designed for targets genes were listed in Table S4 (see Supplementary Table S4). To determine the expression profiles of these genes in different development stages, body parts, and wing morphs, we collected the following for RNA isolation: 30 of each developmental stage (first instar nymph, second instar nymph, third instar nymph, fourth instar nymph, and adult) of apterous and alate aphids; 30 of each of the body parts (head, thorax, and abdomen) from fourth instar winged-nymphs, apterous and alate adults; and 30 wing buds from fourth instar winged-nymphs and alate adults. RNA was extracted as described for the DGE library preparation and sequencing with 4 biological replicates. The first strand cDNA was synthesized from 500 ng of DNA-free RNA using the PrimerScript^®^ RT Reagent Kit Perfect Real time Kit (Takara, Dalian, China) according to the manufacturer instructions. Briefly, all reaction volumes were 10 μL and contained 500 ng of RNA, 200 pmol of Random hexamers, 2 μL of reverse transcription buffer, 0.5 μL of PrimerScript^®^ RT Enzyme Mix I and RNase free H_2_O. The reverse transcription reaction was performed on a C1000^TM^ Thermal Cycler (Bio-Rad, Hercules, CA) at 37 °C for 15 min followed by 85 °C for 5 s. The resulting cDNA were stored at −20 °C until further use.

Expression reactions of all genes were performed using an Mx3000P thermal cycler (Agilent Technologies, Inc., Wilmington, NC). Each 20 μL reaction mixture containing 10 μL of iQ^TM^ SYBR Green Supermix (BIO-RAD, Hercules, CA), 7 μL nuclease-free water, 1 μL of each gene-specific primer (0.2 mM) and 1 μL of the first strand cDNA. The PCR parameters were as follows: 95 °C for 120 s, then 40 cycles at 95 °C for 30 s and 60 °C for 30 s, a final cycle of 60 °C for 30 s and 95 °C for 30 s. The elongation factor-1 alpha (*EF1α*) was used as an internal control^53^ and the relative expression of genes were calculated using the 2^−△△*Ct*^method^54^. The data was analyzed using one-way ANOVA followed by Tukey’s honestly significant difference test (*P* < 0.05) to determine the significant differences among various developmental stages and body parts (head, thorax, and abdomen). Student’s *t*-test was used to separate the means between the wing of N4-WD and alate adults.

### dsRNA synthesis and plant-mediated RNAi

RNAi was used to study the functions of lipid synthesis and the degradation gene (*KAT*) and glycogen related genes (*PEPCK* and *GP*) and the effect on wing development in *T. citricida*. The unique nucleotide regions of the genes were selected for specific dsRNA synthesis. The primers used to synthesize dsRNA are listed in Table S4. All of the reagents and enzymes used for the dsRNA synthesis were from the TranscriptAid T7 High Yield Transcription Kit (Thermo Scientific, Wilmington, DE). The size of the dsRNA products was confirmed by electrophoresis on a 1% agarose gel and the final concentration of dsRNA was 1500 ng/μL.

We developed a method to silence gene activity by dsRNA feeding through a citrus stem based on the methods of RNAi used in *Bemisia tabaci*^55^ and *Sogatella furcifera*^56^ with slight modification. Briefly, an 8-cm-long citrus stem was detached from the citrus seeding and inserted into a 250-μL PCR tube containing 200 μL of dsRNA. Then the tube containing the dsRNA and stem was transferred into a 50 ml plastic tube. Thirty fourth instar winged-nymphs were collected at the same time of day as above description and released onto the stem and the aphids were reared under conditions described above. After feeding with dsRNA for 72 h, morphological changes were observed and photos were taken using a Leica M165C microscope (Leica Microsystems, Wetzlar, Germany). Total RNA was isolated from surviving aphids after feeding for 72 h and the levels of transcripts were measured using qRT-PCR as described above. Four biological replicates were performed for each sample and ds*GFP* was used as a control. One-way ANOVA followed by Tukey’s honestly significant difference test (*P* < 0.05) was used to determine the significant differences among treatments.

## Additional Information

**How to cite this article**: Shang, F. *et al*. Differential expression of genes in the alate and apterous morphs of the brown citrus aphid, *Toxoptera citricida*. *Sci. Rep.* **6**, 32099; doi: 10.1038/srep32099 (2016).

## Supplementary Material

Supplementary Information

Supplementary dataset

## Figures and Tables

**Table 1:** Sequence summary of the Illumina sequencing from *Toxoptera citricida* transcriptome.

Sequencing	
Total number of reads	41,250,684
Total number of clean reads	37,182,535
Total clean nucleotides (bp)	7,510,872,070
Q_30_ percentage (%)	96.17
GC percentage (%)	41.97
Number of contigs	1,199,882
Length of contigs (bp)	85,765,139
N50 length of contigs (bp)	101
Number of unigenes	44,199
Length of unigenes (bp)	33,087,880
Mean length of unigenes (bp)	749
Unigenes annotations against NR	25,682
Unigenes annotations against Swiss-Prot	18,070
Unigenes annotations against KEGG	10,522
Unigenes annotations against GO	12,189
Unigenes annotations against COG	11,052

**Table 2:** The top twenty enriched metabolic pathways among highly expressed transcripts (HETs) in alate adults.

Pathway ID	Description	HETs	P-value	Q-value
ko01100	Metabolic pathways	33	0.01704447	8.59E-02
ko00071	Fatty acid metabolism	10	1.62E-08	1.96E-06
ko05164	Influenza A	9	0.000529084	1.12E-02
ko05130	Pathogenic Escherichia coli infection	8	2.69E-05	1.61E-03
ko05134	Legionellosis	7	3.98E-05	1.61E-03
ko00280	Valine, leucine and isoleucine degradation	7	0.000159331	4.82E-03
ko04145	Phagosome	7	0.006746751	4.30E-02
ko05162	Measles	6	0.000596582	1.12E-02
ko00020	Citrate cycle (TCA cycle)	6	0.000738194	1.12E-02
ko00564	Glycerophospholipid metabolism	6	0.001874586	2.27E-02
ko04144	Endocytosis	6	0.09049112	3.22E-01
ko04612	Antigen processing and presentation	5	0.002141193	2.36E-02
ko05020	Prion diseases	5	0.003264736	3.29E-02
ko04540	Gap junction	5	0.004379756	3.79E-02
ko05145	Toxoplasmosis	5	0.005449289	4.22E-02
ko03320	PPAR signaling pathway	5	0.006048017	4.22E-02
ko04530	Tight junction	5	0.1223737	3.54E-01
ko05169	Epstein-Barr virus infection	5	0.2186322	4.99E-01
ko04141	Protein processing in endoplasmic reticulum	5	0.2950153	5.58E-01
ko04910	Insulin signaling pathway	4	0.2388871	5.26E-01

**Table 3:** The top ten highly expressed transcripts in alate adults of *T. citricida*.

Gene ID	Annotation	Abbreviation	Change fold
c12058.graph_c0	Sodium- and chloride-dependent creatine transporter 1-like isoform 1	*SCDCT*	8.43
c11063.graph_c0	3-ketoacyl-CoA thiolase, mitochondrial-like	*KAT*	8.07
c9682.graph_c0	ATP-binding cassette sub-family G member 4-like isoform 1	*ABCG4*	7.74
c14944.graph_c0	Trifunctional enzyme subunit beta, mitochondrial-like	*β-TE*	5.76
c15715.graph_c0	Short-chain specific acyl-CoA dehydrogenase, mitochondrial-like	*SCAD*	5.54
c15078.graph_c0	Trifunctional enzyme subunit alpha, mitochondrial-like	*α-TE*	5.31
c15087.graph_c0	Phosphoenolpyruvate carboxykinase [GTP]-like	*PEPCK*	5.17
c15313.graph_c0	Hydroxysteroid dehydrogenase-like protein 2-like	*HSD*	4.72
c13625.graph_c0	E3 ubiquitin-protein ligase TRIM23-like	*E3-TRIM23*	4.31
c11587.graph_c1	Glycogen phosphorylase-like isoform 2	*GP*	4.29
