# Supplementary material for: Differential expression of genes in the alate and apterous morphs of the brown citrus aphid, Toxoptera citricida
Source: Sci Rep. 2016 Aug 31;6:32099. doi: 10.1038/srep32099 (PMC5006003; doi:10.1038/srep32099)
Supplement: Supplementary Information [file srep32099-s1.pdf]

Supplementary Section

Differential expression of genes in the alate and apterous morphs of the brown citrus aphid, *Toxoptera citricida*

Feng Shang[1], Bi-Yue Ding[1], Ying Xiong[1], Wei Dou[1], Dong Wei[1], Hong-Bo Jiang[1], Dan-Dan Wei[1], and Jin-Jun Wang[1*]

[1]Key Laboratory of Entomology and Pest Control Engineering, College of Plant Protection, Southwest University, Chongqing 400716, China.

Correspondence: Dr. Jin-Jun Wang, College of Plant Protection, Southwest University, Chongqing 400715, P. R. China. E-mail: wangjinjun@swu.edu.cn; jjwang7008@yahoo.com Tel: (86)-23-68250255; Fax: (86)-23-68251269

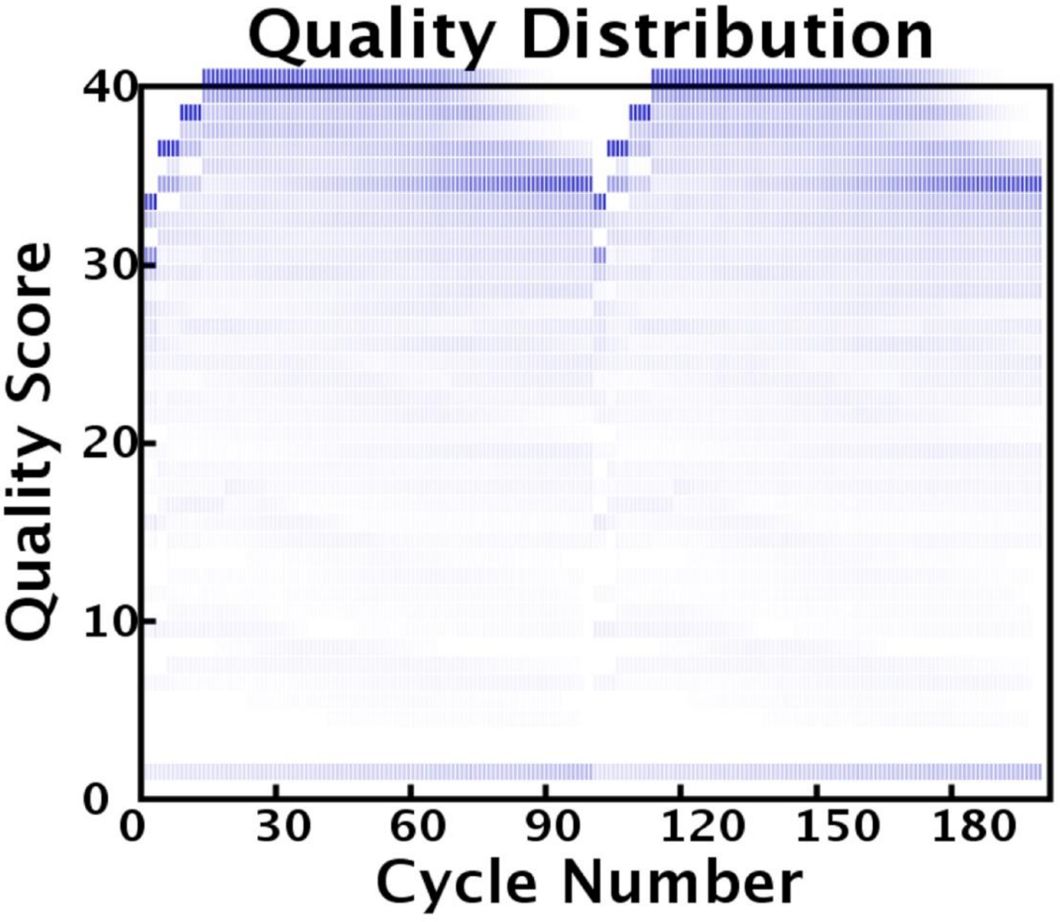

Fig. S1. The quality of clean data of transctiptome of *Toxoptera citricdia*. The X- axis represents base position of reads, and the Y-axis represents mean mass of each cycle.

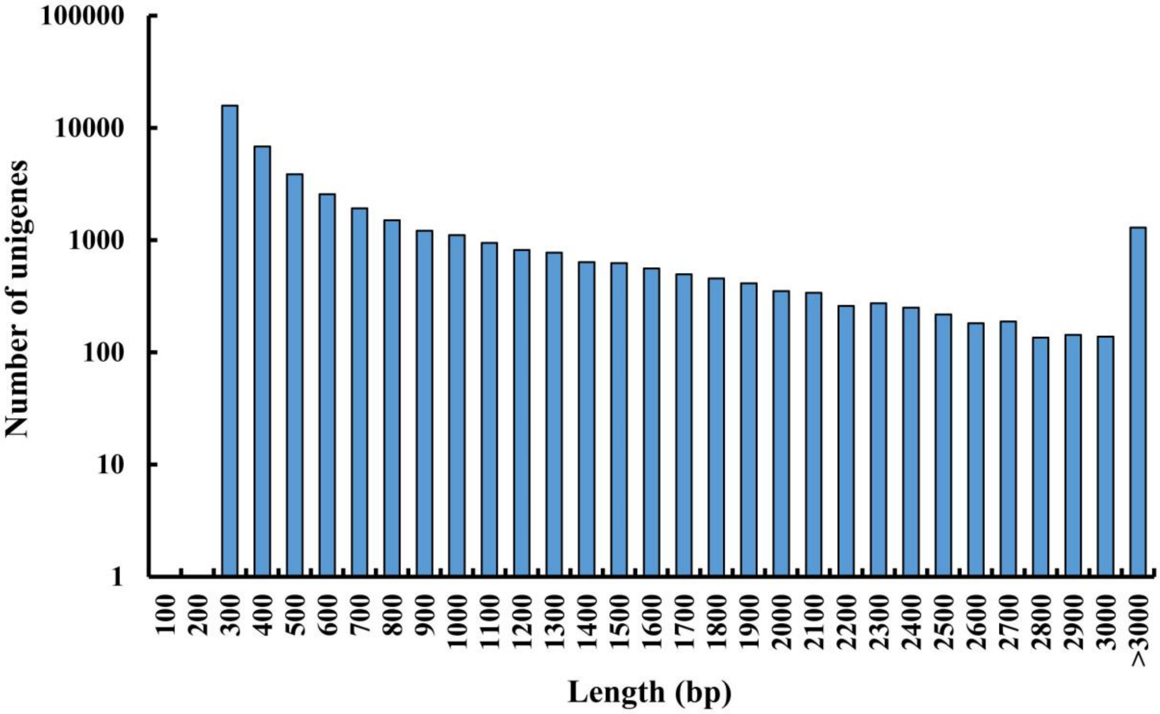

Fig. S2. Length distributions of the *de novo* assembly for unigenes. The length distribution of unigenes were counted with an interval of every 100 bp from 200 bp to 3000 bp. Each number in the x-axis indicates a region of sequence length covering 100 bp, for example, ‘‘300’’represents a region of sequence length [200, 300).

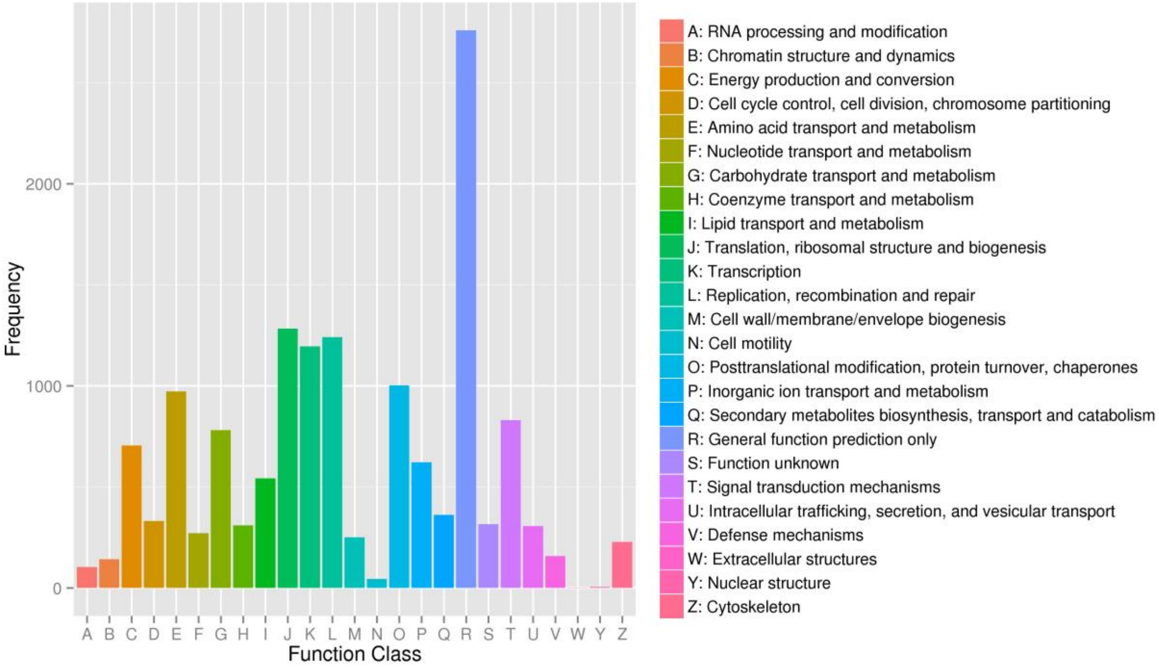

Fig. S3. Classification of the clusters of orthologous groups (COG) for the transcriptome of *Toxoptera citricida*. 11,052 sequences had a COG classification among the 26 categories.

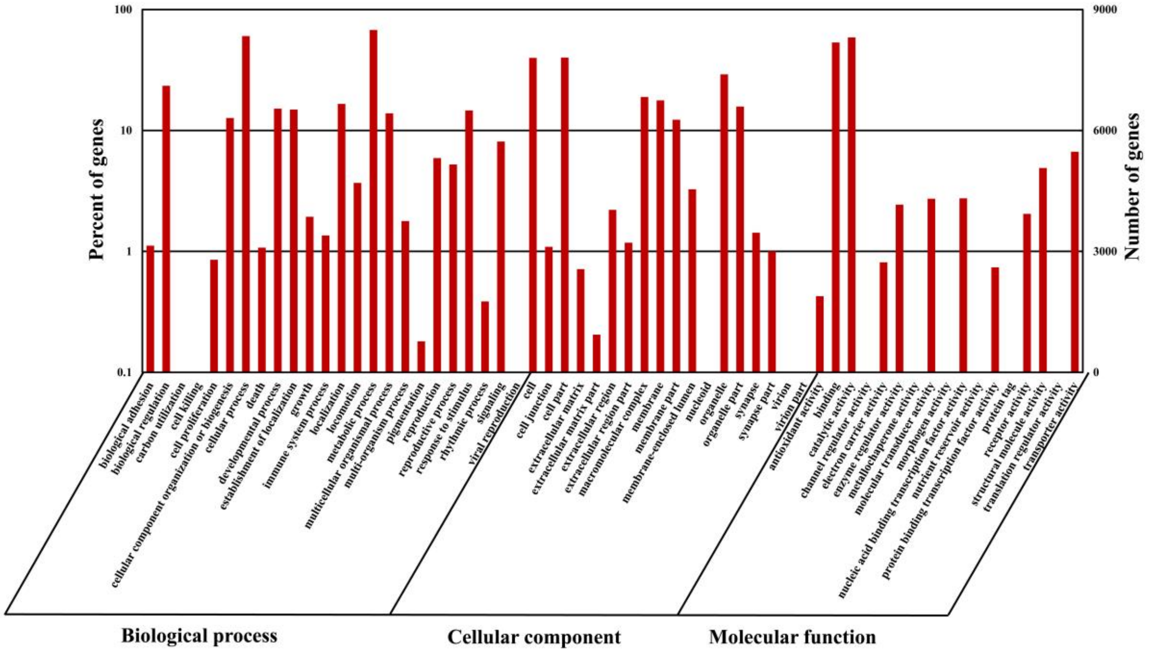

Fig. S4. Classification of the gene ontology (GO) for the transcriptome of *Toxoptera citricida*. 12,189 sequences were annotated within the GO database and summarized in three categories: Biological process, Cellular component and Molecular function. The left and right Y-axis indicate the percentage and number of genes in each category, respectively.

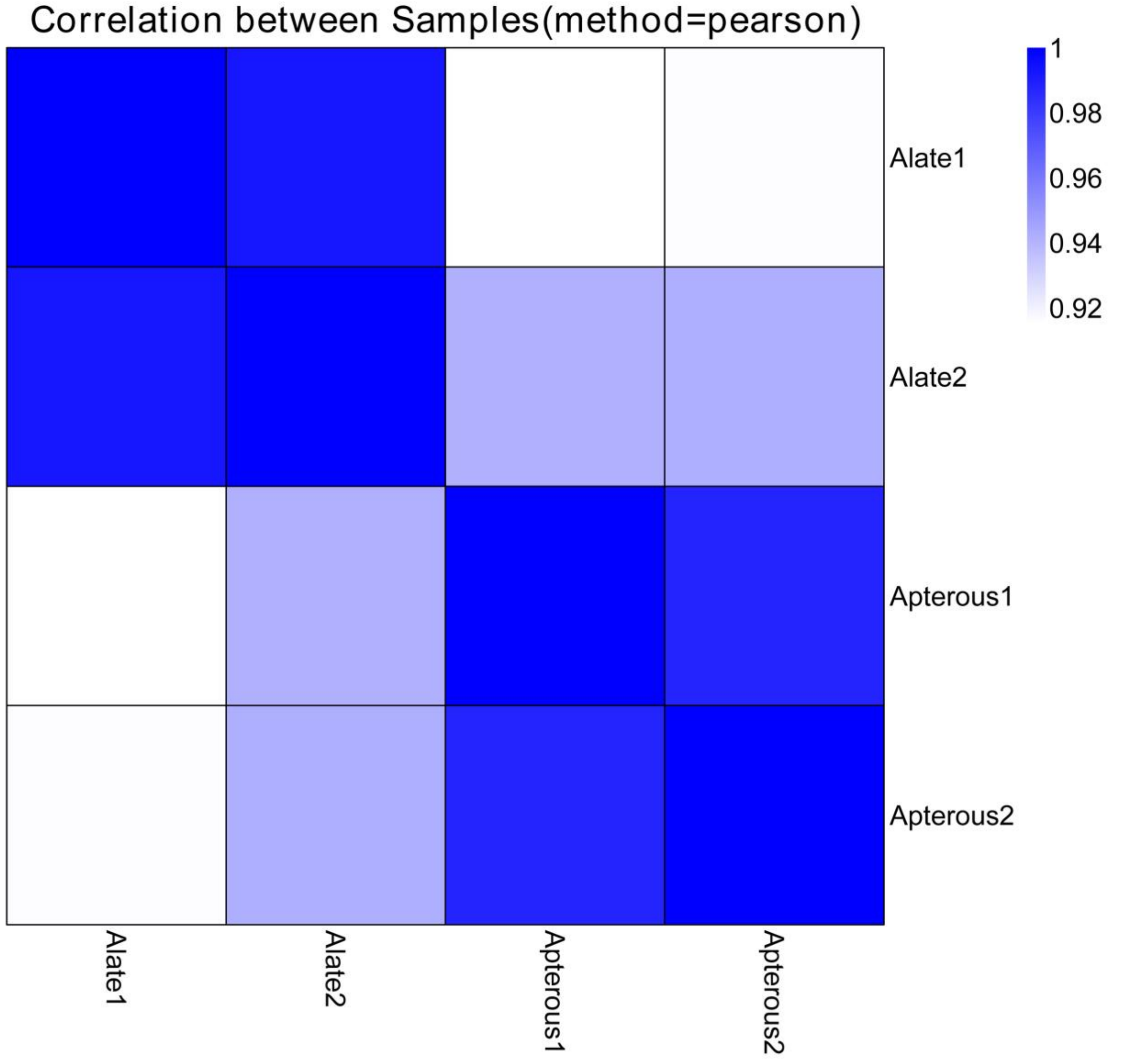

Fig. S5. Heatmap of correlation between each two samples (Alate1 versus Alate 2, and Apterous1 versus Apterous2) (method=pearson).

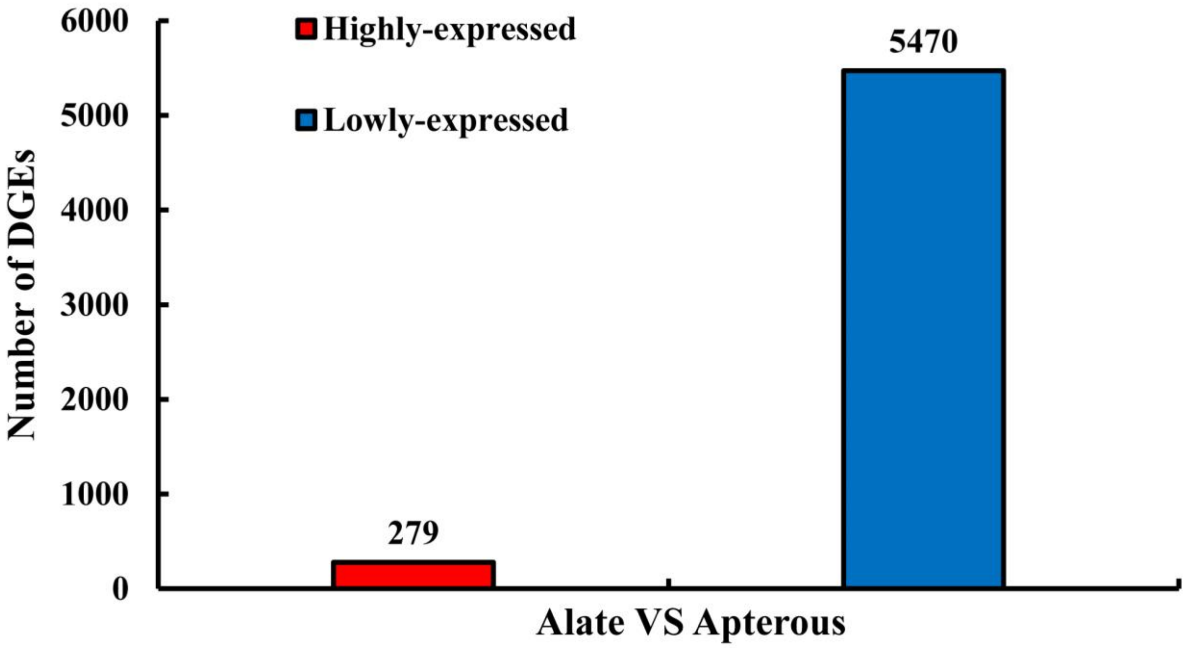

Figure S6. Summary of differentially expressed genes in pairwise comparisons between alate and apterous adults of *Toxoptera citricida*. 279 unigenes were highly expressed in alate adult and 5,470 unigenes were lowly expressed.

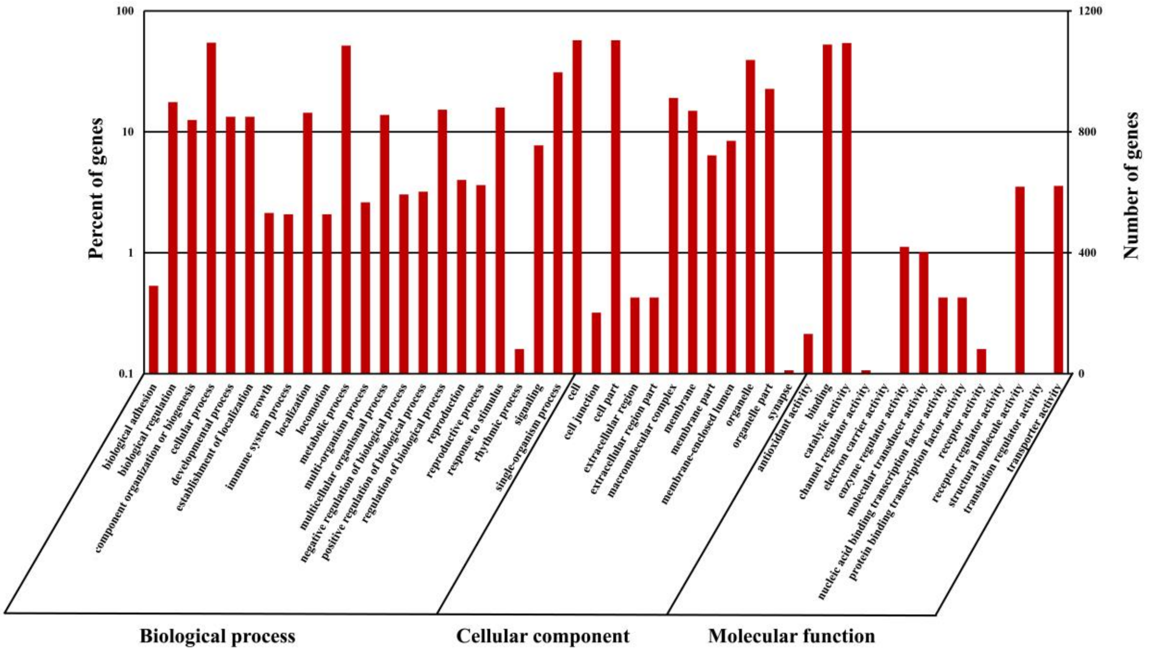

Fig. S7. Classification of the gene ontology (GO) of transcripts differentially expressed between alate adults and apterous adults of *Toxoptera citricida*. 5,749 differentially expressed transcripts were annotated within the GO database and summarized in three categories: Biological process, Cellular component and Molecular function. The left and right Y-axis indicate the percentage and number of genes in each category, respectively.

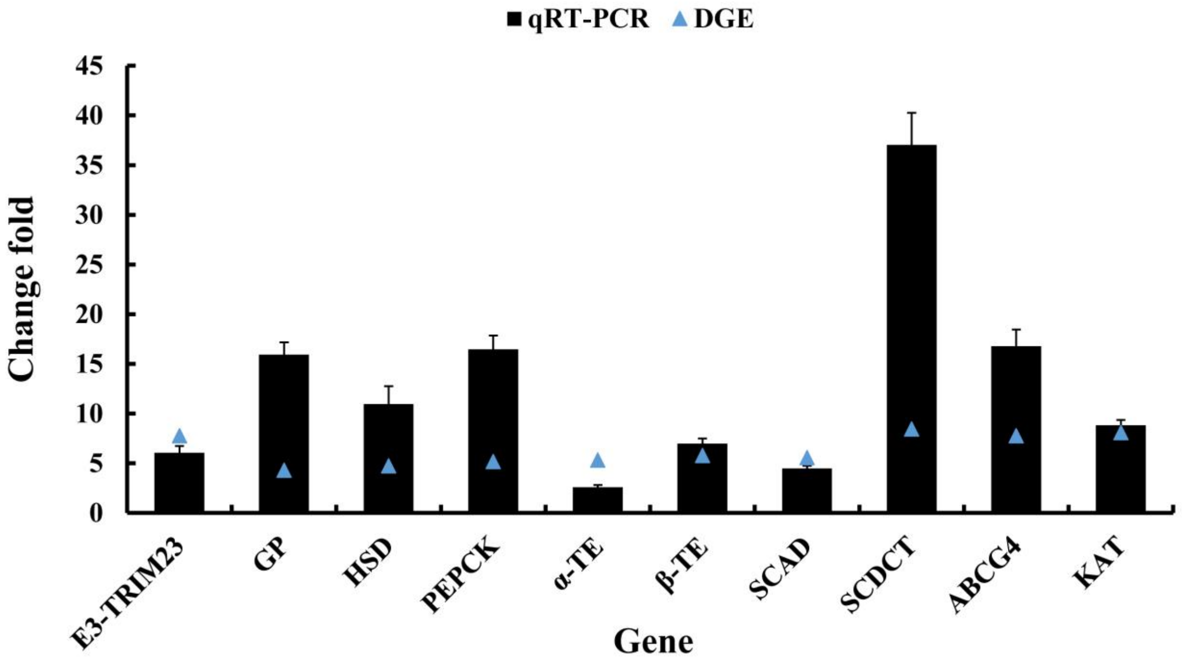

Fig. S8. RT-qPCR validation of top 10 up-regulated genes in alate adults for DGE results. Data are means ± SE of four biological replications. The relative expression was calculated based on the value of the apterous adults expression which was ascribed an arbitrary value of 1. The elongation factor-1 alpha (*EF1α*) was used as a reference gene and the relative expression of genes were calculated using the 2^{-Ct} method
